# Electrocardiographic Changes and Arrhythmia in Fabry Disease

**DOI:** 10.3389/fcvm.2016.00007

**Published:** 2016-03-24

**Authors:** Mehdi Namdar

**Affiliations:** ^1^Service de Cardiologie, Hôpitaux Universitaires de Genève, Geneva, Switzerland

**Keywords:** Fabry disease, arrhythmia, electrocardiography, lysosomal storage diseases, sphingolipidoses

## Abstract

Fabry disease is an X-chromosome-linked lysosomal storage disease characterized by a deficient activity or, in most males, absence of the enzyme α-galactosidase A (a-Gal A) leading to systemic, primary lysosomal accumulation of globotriaosylceramide (Gb3) (1). Recent literature refers to an overall birth prevalence of 1:40,000–170,000; however, such data do not allow an estimation on an *actual* patient number suffering from Fabry disease (2). Multisystem morbidity commonly develops in childhood and, with progression of the disease, life-threatening complications often occur in adulthood, including renal failure, cardiovascular dysfunction, neuropathy, and stroke (3–6). Life expectancy is reduced by an average of 15 years in female patients and 20 years in male patients (7, 8). The pathognomonic Gb3 accumulation has been repeatedly observed over the past decades by many groups in vascular endothelial and smooth muscle cells, cardiomyocytes, cardiac conduction tissue, and valvular fibroblasts (3). Although incompletely described, it is likely that inflammatory and neurohormonal mechanisms are involved in subsequent cellular and vascular dysfunction, leading to tissue ischemia, hypertrophy, and fibrosis (9). Furthermore, recently published works on cardiomyocyte dysfunction and conduction tissue involvement have suggested that cardiac dysfunction may reflect increased myocardial nitric oxide production with oxidative damage of cardiomyocyte myofilaments and DNA, causing cell dysfunction and death, and accelerated conduction with prolonged refractoriness and electric instability (10, 11).

## Cardiac Manifestations

**Figure 1:**
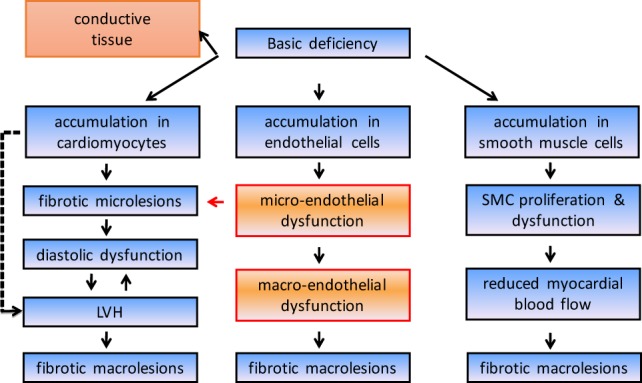
**Simplified course disease pathogenesis in Fabry disease**. LVH, left ventricular hypertrophy.

Cardiac involvement of the disease has been recognized as a common end-organ complication since the earliest reports and is an undisputed driver of morbidity and mortality in Fabry disease. Intracellular accumulation of Gb3 leads through complex cellular cascades and mechanisms that are still not fully explained to irreversible cardiac damage (Figure 1). As a consequence, significant morbidity as well as early death due to heart failure and, most importantly, life-threatening ventricular arrhythmias may result (12). Left ventricular hypertrophy is a key feature and occurs in up to 50% of males and one-third of females (4). In most cases, left ventricular hypertrophy is concentric; however, an asymmetrical variety with septal thickening and posterior wall fibrotic thinning may be present in an advanced stage of the disease. Mild left ventricular diastolic dysfunction is seen early in the disease process and progresses to severe forms in later stages. Right ventricular hypertrophy is also common and may progress to right ventricular dilatation (13). The fibrotic process in Fabry cardiomyopathy starts with intramural, and later transmural, involvement that is invariably present in the basal posterolateral segments (14). However, although myocardial accumulation of Gb3 begins very early in life, left ventricular hypertrophy most commonly manifests itself only decades later, at an average age of 32 years in men and 40 years in women (12). This suggests that the detection of left ventricular hypertrophy might not be suitable for the recognition of *early* stages of the disease. Furthermore, it has become evident that the severity of baseline left ventricular hypertrophy and fibrosis determines the cardiac outcome with enzyme replacement therapy and that the best treatment outcomes can be obtained when treatment is started early.

## Conduction Abnormalities and Arrhythmia Burden in Fabry Disease

**Figure 2:**
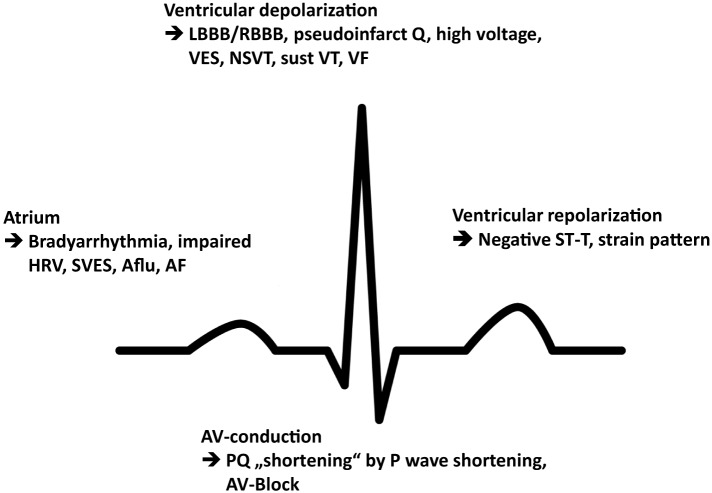
**Schematic illustration of the most prevalent conduction abnormalities and arrhythmia**. HRV, heart rate variability; SVES, supraventricular extrasystole; Aflu, atrial flutter; AF, atrial fibrillation; LBBB, left bundle branch block; RBBB, right bundle branch block; VES, ventricular extrasystole; NSVT, non-sustained ventricular tachycardia; sust VT, sustained ventricular tachycardia; VF, ventricular fibrillation.

Commonly reported arrhythmia and electrocardiographic findings include bradycardia and PQ-interval shortening, which has been shown to be due to shortening of the P-wave duration and one of the first signs of cardiac involvement (15) (Figure 2). Further abnormalities include PQ-interval prolongation, repolarization abnormalities, and atrioventricular block. Of note, increasing age has been demonstrated to be associated with PQ- and QRS-interval prolongation and left QRS axis deviation as well as a progressive sinus and atrioventricular node disease, necessitating a close monitoring for bradyarrhythmias and the implantation of a pacemaker (16). Accordingly, prolongation of the PQ interval, whether shortened or within normal limits at baseline, is a very common finding in the natural history of FD patients, more likely reflecting a progressively increasing disease burden and age-related degenerative processes. The fact that any change in PQ interval may be found at any stage of the disease may only be addressed with speculative explanatory approaches and include the hypothesis that the process of intracardiac Gb3 deposition and treatment response rather follows stochastic rules and may, for instance, start in the atrioventricular perinodal tissue before reaching a homogenous and thus stationary phase of accumulation in later stages of the disease, when triggered cascades of hypertrophy, proliferation, and fibrosis on a cellular–molecular level overrule the importance of Gb3 storage and its direct effects *per se*.

A recent retrospective chart review of 19 patients on ERT also revealed a high utilization of antibradycardic device therapy and suggested that sudden cardiac death in Fabry cardiomyopathy may rather be related to bradycardia than to (in this report) an uncommonly observed significant ventricular arrhythmia (17), which, after all, remains the most life-threatening condition (18, 19). Longitudinal studies suggest that arrhythmias (including atrial fibrillation and ventricular tachycardia) occur in 27–42% of male and 27% of female patients with Fabry disease (14). Of note, all these factors can be encountered in patients with preserved left ventricular function and in the absence of left ventricular hypertrophy or valve disease (20, 21). While different authors have suggested that the prevalence of paroxysmal atrial fibrillation is up to four times greater in Fabry patients than in the general population, such numbers have to be treated with caution (12, 19). Most of the included patients displayed an increased left atrial diameter, diastolic dysfunction, and increased left ventricular wall thickness, all being predicting factors for atrial fibrillation. Prevalence numbers for arrhythmic episodes obviously increase from the onset of overt Fabry-associated cardiopathy in an exponential course with left ventricular remodeling and hypertrophy, elevated left ventricular and atrial pressures, interstitial fibrosis, and, finally, dilatative cardiomyopathy in end stages of the disease (22).

## Value of the Electrocardiogram in Diagnosis

Nevertheless, over all these years, the use of the electrocardiogram remained somehow neglected and retained for the measurement of a shortened or prolonged PQ interval. Changes of the PQ interval have been reported in case studies and observational data from registries and may be the result of different phenomena (23, 24). However, whether abnormalities in the PQ-interval duration were of diagnostic value in patients with Fabry disease or if they correlated with specific clinical or echocardiographic findings remained unclear. As a matter of fact, a first study “demystified” electrocardiography in Fabry disease and reported the relatively low prevalence (14%) of atrioventricular conduction abnormalities in a large cohort (*N* = 207) of patients newly diagnosed with Fabry disease, demonstrating that the short PQ interval cannot be considered a robust and sensitive sign for the diagnosis of this disease (25). Of note, no significant differences in atrioventricular conduction comparing patients with and without left ventricular hypertrophy were found, suggesting that neither PQ-interval shortening nor atrioventricular block showed any correlation with the severity of the cardiac involvement. These initial findings indicated that the duration of the PQ interval has a low diagnostic yield for early recognition and assessment of disease in patients with a Fabry-related cardiopathy, leading further to a first systematic analysis of *early* electrocardiographic changes in patients *without* signs of left ventricular hypertrophy and diastolic dysfunction (15). Here, accelerated depolarization intervals (shortening of P-wave duration and QRS width), increased repolarization duration (QT and QTc), and more pronounced repolarization dispersion (QTc dispersion and Tpeak–Tend dispersion) could be shown in Fabry patients. These early stage patients had indeed shorter PQ intervals as compared to age and heart rate-matched healthy controls, but PQ intervals were mostly still within the “normal” range (i.e., 120–200 ms) and with similar mean values as in the former analysis (139 ± 29 vs. 131 ± 18 ms) (25). Most importantly, shortening of the P-wave duration was found to be the main contributor to the shorter PQ interval and, while both P-wave and PQ-interval duration differ significantly from healthy subjects, P-wave duration yielded a higher diagnostic performance (92% sensitivity and 80% specificity) (15).

Among different conceivable mechanisms (such as changes in P-wave morphology with changes in heart rate; more synchronous activation of the atria through the sinus node activity preferentially exiting closer to Bachmann’s bundle; and accumulation of Gb3 in and around the crista terminalis) having in theory an accelerated intra-atrial conduction as the common denominator, we think that changes on a cellular or subcellular level might finally yield the explanation for the observed electrocardiographic signs. As a matter of fact, an enhanced conduction velocity has been associated with an increase in the diameter of conducting cells and thus alterations of transmembranous ion currents by many drugs and toxins (26). This suggests that the same may be the case for Gb3, which is known to directly interact with cellular membrane compounds, such as ion channels involved in action potential propagation (9, 27). The reported shorter QRS width further suggested that an enhanced conduction velocity might equally occur within the ventricles. Clinical cardiac electrophysiologic case studies have specifically excluded septal accessory pathways as an underlying cause for the observed electrocardiographic changes in Fabry disease (28, 29). Furthermore, a recent study showed a normalization of the QTc interval, PQ interval and, most interestingly, P-wave duration under enzyme replacement therapy along with a reduced disease burden, suggesting that the observed electrocardiographic changes might, at least to some degree, have a link to the Gb3 storage (30). While it has been shown for *glycogen* storage diseases that similar phenomena are *directly* caused by glycogen storage acting as independent conducting elements within the myocardial tissue in and around the atrioventricular node, none of the investigations could confirm the same for patients with Fabry disease so far (31). It is further noteworthy that an accumulation in and around the atrioventricular node does not explain changes in P-wave duration giving rise to the question whether previous reports on PQ-interval shortening alone in other storage diseases should be revisited with respect to this observation. These generated the hypothesis that electrocardiographic parameters may be of considerable help for an earlier recognition of patients, eventually earlier initiation of enzyme replacement therapy before otherwise irreversible organ manifestations occur and, last but not least, be useful as follow-up parameters during the treatment.

However, as various electrocardiographic parameters change with macroscopic myocardial changes (QRS width and repolarization indices with left ventricular hypertrophy, P-wave alterations with left atrial enlargement), an assessment of their value in the differential diagnosis of hypertrophic cardiopathies has been performed (32). As expected, mean left atrial size in Fabry patients with left ventricular hypertrophy was almost double when compared with early stage patients. Accordingly, these enlarged left atrial dimensions might have outbalanced presumable shorter PQ interval and P-wave durations in Fabry patients and thus impaired their diagnostic value. To overcome this shortcoming, the authors chose PQ interval minus P-wave duration in lead II (Pend – Q) as a more robust measurement for atrioventricular conduction. It turned out to have an even higher diagnostic performance for the recognition of Fabry disease as compared to the commonly used PQ interval. Furthermore, a two-step approach combining QTc duration with the above-discussed measure showed a high diagnostic performance for the differentiation of Fabry disease from amyloidosis, and a novel index based on these parameters proved very useful for the differentiation of the two entities Fabry and amyloidosis from hypertensive heart disease, familiar hypertrophic cardiomyopathy, and left ventricular hypertrophy owing to significant aortic stenosis.

## Value of the Electrocardiogram in Disease Staging

Naturally, the thorough investigation of baseline electrocardiographic parameters and their diagnostic value in the recognition of early-stage and differentiation of late-stage Fabry patients begs the question whether these and possibly other parameters might have any value in disease *staging*.

Electrocardiographic changes in a large group of patients with Fabry disease in different disease stages have recently been investigated. Here, the main findings were ST- and T-alterations (ST-declines or elevation; T-wave inversion) giving a clue toward myocardial fibrosis. As a matter of fact, these alterations, mainly found in the lateral leads V5 and V6, fit very well with the region where late enhancement (a sign for focal fibrosis) in magnetic resonance tomography can be seen first. ST- and T-alterations have already been reported in patients with Fabry disease. However, they have been rather misinterpreted as infarct associated lesions in the past since macrovascular coronary artery disease is very rarely encountered in these patients (24, 33). While the detection of fibrotic areas by one single electrocardiographic parameter did not emerge feasible, it is nevertheless reasonable to state that replacement fibrosis is very unlikely when no ST- or T-alterations are observed (34).

The importance of this finding is manifold. First of all, in “electrocardiography negative” Fabry patients, the cost and time consuming process of a cardiac magnetic resonance tomography might not be necessary. Second, as the presence of fibrosis evidently plays a crucial role in terms of a higher incidence of ventricular arrhythmias and poor prognosis in patients with ischemic and non-ischemic cardiopathies, these findings might have a major impact on further diagnostic and therapeutical strategies (35, 36). Different mechanisms are considered to be involved in the pathogenesis of ventricular arrhythmias particularly in the presence of fibrosis. While in ischemic cardiopathies, the main mechanism is assumed to be scar-based reentry, the arrhythmogenic substrate in non-ischemic cardiopathies has been demonstrated to be represented by an increased myocardial collagen content and regional fibrosis (37). Moreover, electrophysiological studies have revealed that patients with sustained ventricular tachycardia show a greater degree of myocardial fibrosis than patients without arrhythmias and that the basal electrocardiographic and intracardiac electrogram abnormalities corresponded very well to the site of origin of the these tachycardias (38). Accordingly, a number of such electrocardiographic abnormalities, invariably coming along with micro- and macroscopic myocardial changes, have been linked to potentially life-threatening reentrant ventricular arrhythmias (39, 40) and shown to be prevalent not only in patients with overt left ventricular hypertrophy but also in an early stage of the disease (15, 32). These and recently published data indicate that the observed electrocardiographic surrogates in conjunction with a fibrotic substrate may be associated with the increased propensity of Fabry patients to develop ventricular arrhythmias (41).

Unfortunately, the prevalence of life-threatening arrhythmia in patients with Fabry disease is not well documented so far. This might be due the paucity of studies involving large populations. Non-sustained ventricular tachycardia has been reported to occur in 38% of men with Fabry disease aged more than 50 years in a study with 78 consecutive patients. While none of these patients had evidence of coronary heart disease as an underlying cause, some already had advanced left ventricular hypertrophy (and possibly fibrotic areas) and might therefore have been susceptible to ventricular arrhythmias (19). Results from the International Fabry Outcome Survey with 714 patients depicted the prevalence of palpitations or documented arrhythmias (not further specified) as 15 and 21%, respectively, in untreated men and women. The average age of these two groups was 26 and 45 years, respectively. Although these numbers suggest that arrhythmias might occur earlier than the development of left ventricular hypertrophy, it is important to note that the prevalence of left ventricular hypertrophy even in these two groups was 33 and 21%, respectively (12). However, an earlier analysis demonstrated that QTc prolongation and pronounced repolarization abnormalities are present before echocardiographic signs of left ventricular hypertrophy are detectable (15). These data indicate that conduction abnormalities do not exclusively occur as a result of the latter but may result from a Fabry-specific disease process and that the temporal as well as the causal interrelationship between the development of conduction abnormalities and/or arrhythmias and left ventricular hypertrophy with or without fibrosis remain unclear.

## Outlook and Future Perspectives

Despite all efforts over the past decades for a better understanding of disease mechanisms in Fabry disease, many questions and unmet needs remain with regards to a satisfying management of affected patients.

To begin with, the presentation and clinical course of this disease is very heterogeneous. Although its progressive character with life-threatening multiorgan manifestations develops generally during the third and fourth decade of life, many symptoms of the disease can occur before 10 years of age, frequently resulting in a mean time between the onset of symptoms and correct diagnosis in these patients of over 15 years, emphasizing the importance of an increased awareness. As enzyme replacement therapy is available, earliest possible recognition and treatment of these patients and family members is of utmost importance. Although substantial progresses have been made in the past years studying histopathological changes associated with Gb3 accumulation in animal model studies using GLA-knock-out mice, they remain, for the time being, of limited value for Fabry disease-associated cardiomyopathy, since they lack of lysosomal Gb3 storage in vascular endothelial cells and cardiomyocytes (42).

Several electrocardiographic characteristics have been described to occur not only early in the disease course but also before morphologic alterations develop, as most of the included patients had normal echocardiograms. Whether these electrocardiographic abnormalities have a more diagnostic value in patients with Fabry disease and whether they correlate with specific clinical or echocardiographic findings remains elusive and are a matter of current investigations. In this regard, electrophysiologic studies in patients with a confirmed diagnosis in any stage of the disease are needed.

Furthermore, a prospective cohort study to clarify the prognostic significance of conduction abnormalities and arrhythmias, their development under enzyme replacement therapy, and the causes of mortality does not exist to date. Therefore, in contrast to the prognostic significance of left ventricular hypertrophy, the question remains open as to what extent arrhythmias are of clinical or even prognostic significance that should be taken seriously in Fabry disease. Finally, despite many questions left, these parameters may identify patients at a high risk to justify the use of an implantable cardioverter defibrillator in order to treat malignant ventricular arrhythmias, one of the most dreaded complications of Fabry disease.

## Author Contributions

MN conceptualized and wrote the manuscript.

## Conflict of Interest Statement

MN has received financial support from Genzyme/Sanofi and Shire: travel grants, research grants, speaker’s fees, and honoraria for advisory board activities.
